# Immunohistochemistry for Prostate Biopsy—Impact on Histological Prostate Cancer Diagnoses and Clinical Decision Making

**DOI:** 10.3390/curroncol28030197

**Published:** 2021-06-09

**Authors:** Philipp Mandel, Mike Wenzel, Benedikt Hoeh, Maria N. Welte, Felix Preisser, Tahir Inam, Clarissa Wittler, Clara Humke, Jens Köllermann, Peter Wild, Christoph Würnschimmel, Derya Tilki, Markus Graefen, Luis A. Kluth, Pierre I. Karakiewicz, Felix K.-H. Chun, Andreas Becker

**Affiliations:** 1 Department of Urology, University Hospital Frankfurt, Goethe University Frankfurt, 60590 Frankfurt, Germany; philipp.mandel@kgu.de (P.M.); Benedikt.hoeh@kgu.de (B.H.); maria-noemi.welte@kgu.de (M.N.W.); felix.preisser@kgu.de (F.P.); medizintahir@googlemail.com (T.I.); Clarissa.Wittler@kgu.de (C.W.); Clara.Humke@kgu.de (C.H.); luis.kluth@kgu.de (L.A.K.); felix.chun@kgu.de (F.K.-H.C.); andreas.becker@kgu.de (A.B.); 2 Cancer Prognostics and Health Outcomes Unit, Division of Urology, University of Montreal Health Center, Montreal, QC H2X3A4, Canada; c.wuernschimmel@uke.de (C.W.); pierre.karakiewicz@umontreal.ca (P.I.K.); 3 Dr. Senckenberg Institute of Pathology, University Hospital Frankfurt, 60590 Frankfurt, Germany; jens.koellermann@kgu.de (J.K.); peter.wild@kgu.de (P.W.); 4 Frankfurt Institute for Advanced Studies (FIAS), 60590 Frankfurt, Germany; 5 Wildlab, University Hospital Frankfurt MVZ GmbH, 60590 Frankfurt, Germany; 6 Martini-Klinik Prostate Cancer Center, University Hospital Hamburg-Eppendorf, 20251 Hamburg, Germany; tilki@uke.de (D.T.); graefen@uke.de (M.G.); 7 Department of Urology, University Hospital Hamburg-Eppendorf, 20251 Hamburg, Germany

**Keywords:** immunohistochemistry, IHC, prostate cancer, radical prostatectomy, ASAP, Gleason Score

## Abstract

Background: To test the value of immunohistochemistry (IHC) staining in prostate biopsies for changes in biopsy results and its impact on treatment decision-making. Methods: Between January 2017–June 2020, all patients undergoing prostate biopsies were identified and evaluated regarding additional IHC staining for diagnostic purpose. Final pathologic results after radical prostatectomy (RP) were analyzed regarding the effect of IHC at biopsy. Results: Of 606 biopsies, 350 (58.7%) received additional IHC staining. Of those, prostate cancer (PCa) was found in 208 patients (59.4%); while in 142 patients (40.6%), PCa could be ruled out through IHC. IHC patients harbored significantly more often Gleason 6 in biopsy (*p* < 0.01) and less suspicious baseline characteristics than patients without IHC. Of 185 patients with positive IHC and PCa detection, IHC led to a change in biopsy results in 81 (43.8%) patients. Of these patients with changes in biopsy results due to IHC, 42 (51.9%) underwent RP with 59.5% harboring ≥pT3 and/or Gleason 7–10. Conclusions: Patients with IHC stains had less suspicious characteristics than patients without IHC. Moreover, in patients with positive IHC and PCa detection, a change in biopsy results was observed in >40%. Patients with changes in biopsy results partly underwent RP, in which 60% harbored significant PCa.

## 1. Introduction

Prostate cancer diagnosis after prostate biopsies, and the subsequent treatment decision making, affect millions of men worldwide yearly [1,2,3]. The detailed pathological results of the prostate biopsy mainly influence treatment decision-making in addition to clinical stage and other parameters such as PSA [2,3,4]. Specifically, biopsy Gleason/ISUP grade in addition to numbers of positive cores and percentage of positive biopsy cores provide clinicians with detailed pathological biopsy information for treatment decision-making, to recommend, for example, active surveillance or definite treatments [2,4,5,6]. However, results of prostate biopsies and final pathologies after treatment with racial prostatectomy can strongly differ and misclassify patients after biopsies, which instead harbored more aggressive disease [7,8,9]. To predict the final pathology report and avoid underestimation of patients’ biopsy results, several nomograms or epidemiological studies have been published [10,11,12,13]. The main goals of the pathological prostate biopsy results are to provide concordance with the final pathological results. Therefore, pathological guidelines recommend immunohistochemistry (IHC) stains to validate/reject prostate cancer diagnosis in suspicious atypical foci [14]. Moreover, IHC stains are recommended to provide additional information about positive cores and/or percentage of positive cores if this information would affect clinical treatment decision making; for example, inclusion for active surveillance [14,15,16,17]. Until now, evidence was lacking with regard to the clinical impact and changes in treatment decision-making after additional performance of IHC for prostate biopsies. Moreover, little is known about final pathologies in patients with additional IHC for prostate biopsies and subsequent changes in treatment due to IHC information [18].

We addressed this void and relied on our prospective institutional prostate biopsy and radical prostatectomy database. We aimed to investigate the effect of IHC in prostate biopsies with regard to clinical treatment decision making and changes in treatment due to positive IHC. We hypothesized that additional IHC performance in prostate biopsies may influence clinicians’ treatment decision making.

## 2. Materials and Methods

### 2.1. Study Population

After approval of the local ethics committee, all patients who received prostate biopsies between January 2017 and June 2020 at the Department of Urology at Frankfurt University Hospital were in the prospective institutional prostate biopsy database and identified and evaluated retrospectively. Indications for performing a prostate biopsy were suspicious characteristics such as a digital rectal examination (DRE), suspicious PSA values (defined as suspicious absolute PSA, PSA velocity, PSA density, or PSA age cut-offs for each individual patient), or suspicion of prostate cancer on MRI, defined as PIRADS ≥ 3. This selection criteria yielded 606 prostate biopsy patients. All biopsies were performed by experienced urologists under a transrectal approach under antibiotic prophylaxis and periprostatic local anesthesia, as recommended and described [2,19]. For systematic biopsy, 12-core biopsies (length of 15–22 mm per core) were obtained with six biopsies per prostate lobe. Additional targeted biopsy was performed with a HiVison, Hitachi Medical Systems ultrasound machine, and at least two cores were taken from each mpMRI lesion ≥ PIRADS 3. Patients were firstly stratified according to the usage of IHC in pathological prostate biopsy results. IHC was performed in accordance with pathological guidelines, which recommend IHC stains to validate/reject prostate cancer diagnosis in suspicious atypical foci or to provide additional information about positive cores and/or core percentage of positive cores.

### 2.2. IHC Stains

After formalin fixation and paraffin embedding, three to four haematoxylin and eosin-stained sections were routinely prepared from each prostate biopsy, as well as an additional unstained section for any additional immunohistochemical studies that may be required. Immunohistochemical analysis was performed using the automated staining platform DAKO Omnis (Dako/Agilent, Santa Clara, CA, USA) on 1–2-µm-thick sections from formalin-fixed paraffin embedded prostate biopsies. The following antibodies were used: p63 (clone DAK-p63, DAKO/Agilent, mouse monoclonal, ready to use), cytokeratin 5/6 (clone D5/16 B4, DAKO/Agilent, mouse monoclonal, ready to use), and high molecular weight cytokeratins (clone 34ßE12, DAKO/Agilent, mouse monoclonal, ready to use) in combination (double staining) with an antibody to Alpha-methyl acyl coenzyme-A racemase (AMACR) (clone 13H4) DAKO/Agilent, rabbit monoclonal, ready to use. The antibodies were configured as FLEX Ready-to-Use (Agilent) and used with the EnVision FLEX visualization system (Agilent, Santa Clara, CA, USA) according to the manufacturer’s instructions for use.

### 2.3. Changes in Biopsy Results and Changes in Clinical Decision Making

**Figure 1:**
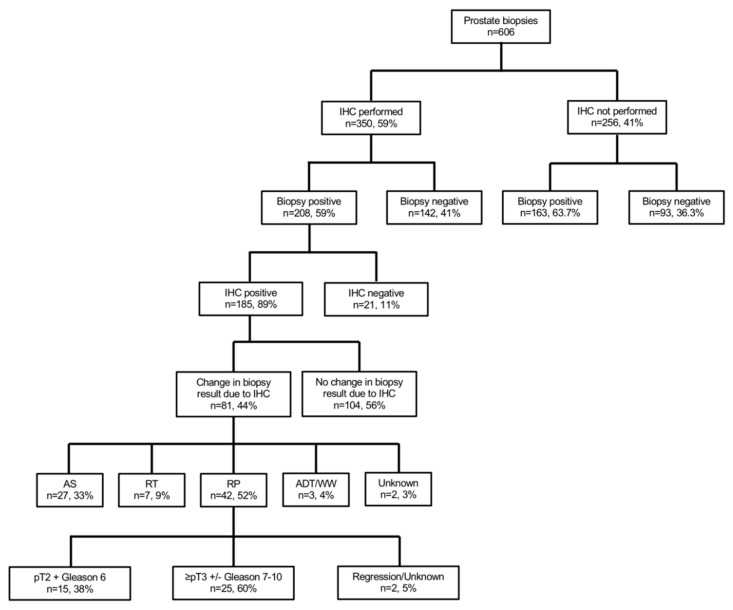
Flow chart depicting the performed subgroup and sensitivity analyses of 606 patients who underwent prostate biopsy at University Hospital Frankfurt between January 2017 and June 2020. Abbreviations: IHC—immunohistochemistry; AS—active surveillance; RT—radiation therapy; RP—radical prostatectomy; ADT—androgen deprivation therapy; WW—watchful waiting.

Patients with pathologically confirmed prostate cancer after prostate biopsies were subsequently stratified according to the performance of IHC (Figure 1). Furthermore, we classified changes after IHC performance as relevant if a change from initial high-grade prostatic intraepithelial neoplasia or atypical small acinar proliferation foci to prostate cancer diagnoses, or a change from unilateral to bilateral prostate cancer, occurred. Moreover, we relied on information from the institutional patient files and the prospective institutional radical prostatectomy database to identify subsequent treatment after additional IHC in prostate biopsies and to identify final pathological results if patients underwent radical prostatectomy. Clinically significant prostate cancer was defined as Gleason score ≥ 7 and/or ≥pT3 stage, as previously reported [20,21].

### 2.4. Statistical Analysis

Descriptive statistics included frequencies and proportions for categorical variables. Means, medians, and interquartile ranges (IQR) were reported for continuously coded variables. The Chi-square test was used for statistical significance in proportions’ differences. The t-test and Kruskal-Wallis test examined the statistical significance of means’ and distributions’ differences. 

Subgroup and sensitivity analyses were made to validate the effect of additional IHC in prostate biopsies in real-world clinical application. All tests were two sided with a level of significance set at *p* < 0.05, and R software environment for statistical computing and graphics (version 3.4.3, Vienna, Austria) was used for all analyses. 

## 3. Results

### 3.1. Descriptive Baseline Characteristics: IHC vs. No IHC

Of 606 patients with prostate biopsy, 350 (58.7%) received additional IHC stains (Table 1, Figure 1). Patients with additional IHC stains at biopsy more frequently harbored non-suspicious DRE (51.1 vs. 42.6%, *p* < 0.01) and PIRADS3 lesions in MRI (28.9 vs. 23.0%, *p* = 0.03). No differences between both groups were observed according to median age at biopsy (66 vs. 67 years), median PSA (7.3 [IQR 5.2–11.9] vs. 8.1 [IQR 5.3–15.8] ng/mL), median number of cores taken at biopsy (13 vs. 13), or repeat biopsies (25.5 vs. 21.9%, all *p* > 0.05). No significant differences were observed in IHC performance in patients with a low PSA <4ng/mL (16.3 vs. 13.3%, *p* = 0.4). Overall, prostate cancer was found in 208 (59.4%) patients with IHC and in 163 (63.7%) patients without IHC (*p* = 0.3). 

According to suspicious prostate cancer characteristics, rates of positive IHC were significantly more often observed in patients with suspicious DRE (88.4 vs. 57.5%) and in patients with PSA ≥4 ng/mL (55.5 vs. 40.4%). Conversely, rates of IHC were more frequently negative in patients with PIRADS 3 lesion (23.1 vs. 9.2%, all *p* ≤ 0.03). 

### 3.2. IHC in Patients with and without Prostate Cancer Detection 

Of 208 patients with prostate cancer detection and IHC stains (Figure 1), IHC was histologically positive in 185 (88.9%) patients (Table S1). Moreover, rates of non-suspicious DRE, median number of positive cores, percentage of positive cores at biopsy, and the median of the maximum tumor infiltration per core between IHC vs. non IHC were 55.3 vs. 39.3%, 5 (IQR 2–7) vs. 6 (IQR 4–10), 40% (IQR 20–60) vs. 50% (IQR 30–80), and 50% (IQR 20–80) vs. 70% (50–90%, all *p* < 0.01), respectively. Moreover, patients with IHC more frequently harbored Gleason 6 (29.8 vs. 4.9%, *p* < 0.01) than patients without IHC. Additionally, rates of Gleason 7 and 8–10 in biopsy were 38.5 vs. 49.1% and 28.8 vs. 44.8% for IHC vs. no IHC, respectively. 

Of 235 patients without prostate cancer detection in biopsy, 142 (60.4%) received additional IHC to rule out prostate cancer (Figure 1). Descriptive characteristics are summarized in Tables S2 and S3.

### 3.3. Changes in Prostate Biopsy Results due to IHC Stains

Of 185 patients with histologically positive IHC and prostate cancer detection (Figure 1), IHC led to a change in prostate biopsy results in 81 (43.8%) vs. 104 (56.2%) patients without any changes in biopsy results (Table 2). Patients with changes in biopsy results had significantly lower PSA (7.1 vs. 9.8 ng/mL), lower percentage of positive cores (20 vs. 50%), and lower maximum tumor infiltration per core (20 vs. 60%, all *p* < 0.01), relative to patients without changes due to positive IHC. Moreover, suspicious DRE and cT2, as well as cT3–4 stages were more frequently in the group without changes in biopsy results. Median number of IHC stains per biopsy did not differ between both groups (4 vs. 4; *p* = 0.06).

Of 81 patients with changes in biopsy results due to positive IHC (Table 3, Figure 1), 55 (67.9%) changed from initial high-grade prostatic intraepithelial neoplasia or atypical small acinar proliferation foci to prostate cancer diagnoses. Moreover, in 26 (32.1%) patients, the change in biopsy results due to positive IHC led to a bilateral prostate cancer diagnoses instead of a unilateral prostate cancer. 

When patient characteristics in patients with changes in biopsy results from precancer lesions to prostate cancer were compared to patients with no IHC performance and negative biopsy, we observed that that patients with negative biopsy and no IHC were younger (63 vs. 69, *p* < 0.01) and underwent less frequently fusion biopsy (40.9 vs. 67.3%, *p* < 0.01).

### 3.4. Treatments of Patients with Changes in Prostate Biopsy due to Positive IHC

Of 81 patients with changes in biopsy results due to positive IHC (Table 3, Figure 1), 42 (51.9%) underwent subsequent radical prostatectomy, 27 (33.3%) underwent active surveillance, and seven (8.6%) radiation therapy as a curative treatment concept. Conversely, three (3.7%) patients underwent androgen deprivation therapy or watchful waiting as a palliative concept. 

Of those patients who underwent subsequently radical prostatectomy, 15 (35.7%) harbored pT2 and Gleason 6 in the final pathology, while 25 (59.5%) patients harbored significant prostate cancer with ≥pT3 and/or Gleason 7–10. Of those 25 patients, 16 (64.0%) initially harbored a unilateral and a change to bilateral prostate cancer due to IHC. Moreover, nine (36.0%) of those patients harbored a high-grade prostatic intraepithelial neoplasia or atypical small acinar proliferation foci initially and had a change to prostate cancer diagnoses due to IHC.

## 4. Discussion

We hypothesized that additional IHC in prostate biopsies may influence pathology results and, therefore, also clinicians’ and patients’ treatment decision making. We tested this hypothesis within our institutional biopsy and radical prostatectomy database and made several noteworthy observations.

First, in patients who received IHC at prostate biopsy and those who did not receive IHC, we made important observations regarding patient characteristics. In total, 59% of all prostate biopsies received additional IHC diagnostics for prostate biopsy. Moreover, IHC was mainly used in patients with lower PSA (albeit not statistically significance, probably mainly due to sample size limitations) and unsuspicious DRE, relative to patients without IHC in prostate biopsies. Moreover, in patients with IHC and prostate cancer diagnoses, positive cores per biopsy and tumor infiltration were lower than in patients without IHC and prostate cancer diagnosis in biopsy. These findings are particularly noteworthy, since IHC was mainly used in patients with the most unsuspicious clinical characteristics, and have to be interpreted in the light that the risk of more aggressive disease increases with specific prostate characteristics such as a high PSA level [22,23]. In consequence, patients with lower but still suspicious PSA and unsuspicious DRE are not only difficult to classify in clinical practice for urologists, but also for pathologists regarding a possible prostate cancer diagnosis. Taken together, it seems that IHC provides a safety tool for pathologists to reject or validate prostate cancer diagnoses and affects the majority of patients. This thesis is also emphasized by the fact that IHC was performed in a high proportion of patients (>47%) in order to rule out prostate cancer diagnoses (negative biopsy), which is also very important to reliably reject the cancer diagnosis.

Second, we also made important observations in the comparison between IHC and non-IHC patients with positive prostate biopsies. Here, similar observations as in the overall cohort of all prostate biopsies were made. Specifically, in the IHC group, the PSA was also lower (8.2 (IQR 5.9–12.9) vs. 9.2 (IQR 5.7–29.6)), albeit not reaching significance, probably due to limitations in sample size. Moreover, DRE was more frequently unsuspicious, and numbers of positive biopsy cores and tumor infiltration were lower in the IHC group. Additionally, patients with prostate cancer diagnosis and IHC harbored less aggressive disease than patients without IHC. Specifically, 30% of IHC patients with prostate cancer exhibited Gleason score 6/ISUP grade 1. Conversely, 5% of patients without IHC and prostate cancer exhibited Gleason score 6/ISUP grade 1. These sensitivity analyses in patients with prostate cancer diagnosis emphasize the additional value of IHC in prostate cancer patients with lower tumor burden and less suspicious clinical characteristics, such as lower PSA and lower rates of DRE. Moreover, these observations are in agreement with current literature and may emphasize the assumption that IHC may help to avoid repeat prostate biopsies since smaller prostate cancer foci can be found easier in the first course of biopsies, relative to patients who did not receive IHC in the biopsy and therefore may exhibited negative prostate biopsy results [24,25,26]. Additionally, it is important to emphasize that IHC was performed in >47% of patients to rule out prostate cancer.

Third, we also revealed important findings according to changes in prostate biopsies due to the additional IHC performance. Of all patients with positive IHC and prostate cancer, IHC performance resulted in 44% of cases in a biopsy change. In two thirds of these patients, application of IHC resulted in a change from a high-grade prostatic intraepithelial neoplasia or atypical small acinar proliferation to prostate cancer diagnoses. In approximately one third, application of IHC changed a unilateral tumor to a bilateral tumor. These findings are particularly relevant, since in atypical small acinar proliferation foci, prostate cancer is found in over 30% in repeat biopsies [27,28]. This underlines the fact that IHC helps to avoid unnecessary repeat biopsy and avoids delayed prostate cancer diagnoses. Additionally, the findings of bilateral prostate cancer diagnosis are noteworthy, since the administration of focal therapies are mostly discussed in single lesion unilateral prostate cancers [29,30,31]. On the other hand, the IHC results did not change the biopsy results in 56% of patients with positive IHC and prostate cancer. This observation questions the rationale that in selected patients, IHC might be avoided with regard to an economic perspective and cost effectiveness [32].

Fourth, we also made important observations according to the treatment of patients with changes in biopsy results due to positive IHC. Specifically, the majority of these patients subsequently underwent radical prostatectomy, while one third underwent active surveillance (*n* = 27, Figure 1). In radical prostatectomy patients, a final pathology of pT2 Gleason 6 was found in 36% (*n* = 15), and ≥pT3 and/or Gleason 7–10 in 60% (*n* = 25). Significant prostate cancer was mainly found in the cohort of patients with initial unilateral prostate cancer which changed to a bilateral cancer due to IHC. These findings are also in an agreement with current literature. For example, Bokhorst et al. described recently in a reevaluation of prostate biopsies with additional performance of IHC, that IHC had a significant impact on treatment decision-making and changed initial treatment plans of patients from active surveillance to active treatments [18]. Those observations emphasize that IHC not only contributes to changes in biopsy results, but also in its clinical application for treatment decision making in daily urological practice. Moreover, an undeniable proportion of radical prostatectomy patients in our cohort with changes in biopsy results due to positive IHC harbored unfavorable tumor characteristics. In consequence, IHC may not only help pathologists to validate or reject prostate cancer diagnoses, IHC may also help to identify patients with risk of non-organ confined disease or unfavorable tumor grade characteristics and patients for active surveillance.

Our study has limitations and needs to be interpreted in its retrospective design. Moreover, sample size limitations might impair statistical significance in some of the analyses, especially in PSA analyses. However, the PSA distribution provided an undeniable trend towards higher PSA in non-IHC patients. Secondly, although prostate biopsies were analyzed by experienced uropathologists, interobserver variability cannot completely be ruled out, nor the decision of whether IHC was performed mainly based on the pathologists’ decision. However, all pathologies were confirmed by an independent second pathologist. Furthermore, due to its study design, our findings cannot give answers about the sensitivity or specificity of IHC in prostate biopsies. Prospective studies are needed to further validate or reject our findings. Finally, unfortunately, no long-term follow-up data or further treatments/pathologies are available for patients who underwent active surveillance after changes in biopsy results due to IHC performance.

Taken together, our findings address several clinically important questions. First, majority of patients receive IHC in prostate biopsies. Second, of all patients with IHC, IHC is positive in the majority of patients, but can also be used to rule out prostate cancer. Third, patients with IHC mostly harbor less suspicious clinical and prostate-specific characteristics than patients without IHC. Fourth, in patients with positive IHC, >40% benefit from a change of the biopsy results. Finally, patients with changes in biopsy results mostly underwent subsequent active treatment with radical prostatectomy and significant prostate cancer was found in 60% of patients.

## 5. Conclusions

Patients with IHC stains mostly harbored less suspicious clinical and prostate-specific characteristics than patients without IHC. Moreover, in patients with positive IHC and PCa detection, a change in biopsy results was observed in >40%. Finally, patients with changes in biopsy results partly underwent active treatment with RP, in which 60% harbored significant PCa. 

## Figures and Tables

**Table 1:** Descriptive analyses of 606 patients who underwent prostate biopsy at University Hospital stratified according to the performance of immunohistochemistry (IHC). Abbreviations: PSA—prostate-specific antigen; DRE—digital rectal examination; GS—Gleason score.

Variable	-	No IHC N = 256 (41.3%)	IHC N = 350 (58.7%)	*p* Value
Age	Median (IQR)	67 (61–72)	66 (60–72)	0.6
Prostate volume	Median (IQR)	48 (35–70)	50 (38–70)	0.4
PSA	Median (IQR)	8.1 (5.3–15.8)	7.3 (5.2–11.9)	0.08
PSA ≥ 4 ng/mL	Yes	222 (86.7)	293 (83.7)	0.4
	No	34 (13.3)	57 (16.3)	
Cores per biopsy	Median (IQR)	13 (12–14)	13 (12–14)	0.06
Positive cores per biopsy	Median (IQR)	3 (0–7)	1 (0–5)	<0.01
Percentage of positive cores	Median (IQR)	50 (30–80)	40 (20–60)	<0.01
Highest tumor infiltration per core	Median (IQR)	60 (15–90)	30 (1–60)	<0.01
DRE	Non-suspicious	109 (42.6)	179 (51.1)	<0.01
	Suspicious	102 (39.8)	86 (24.6)	
cT stage	cT1	109 (42.6)	177 (50.6)	<0.01
	cT2	86 (33.6)	74 (21.1)	
	cT3–4	16 (6.2)	12 (3.4)	
Previous biopsies	Biopsy naive	200 (78.1)	258 (73.7)	0.3
	Repeat biopsy	56 (21.9)	90 (25.7)	
Fusion biopsy	Yes	131 (51.2)	211 (60.3)	0.03
	No	125 (48.8)	138 (39.4)	
MRI lesion	PIRADS 1–2	2 (0.8)	3 (0.9)	0.03
	PIRADS 3	23 (9.0)	55 (15.7)	
	PIRADS 4	59 (23.0)	101 (28.9)	
	PIRADS 5	44 (17.2)	48 (13.7)	
	Unknown /no MRI	128 (50.0)	143 (40.9)	
Biopsy positive?	No	93 (36.3)	142 (40.6)	0.3
	Yes	163 (63.7)	208 (59.4)	
IHC	Negative/Unclear	-	165 (47.1)	-
	Positive	-	185 (52.9)	
Change of biopsy result after positive IHC	No	-	104 (56.2)	-
	Yes	-	81 (43.8)	
Gleason Score at biopsy	6	8 (3.1)	62 (17.8)	<0.01
	7	80 (31.0)	80 (22.9)	
	8–10	73 (28.3)	59 (16.9)	
	No PCa	93 (36.3)	142 (40.6)	
	Unknown GS	4 (1.6)	6 (1.7)	

**Table 2:** Descriptive analyses of 185 patients with positive prostate biopsy and histological positive immunohistochemistry (IHC) at University Hospital Frankfurt between 01/2017–06/2020, stratified according to changes in biopsy results due to positive IHC results or not. Abbreviations: PSA—prostate-specific antigen; DRE—digital rectal examination; GS: Gleason score.

Variable	-	Changes in Biopsy Results due to Positive IHC N = 81 (43.8%)	No Changes in Biopsy Results due to Positive IHC N = 104 (56.2%)	*p* Value
Age	Median (IQR)	67 (61–72)	68 (64–74)	0.18
Prostate volume	Median (IQR)	54 (38–74)	45 (35–55)	0.051
PSA	Median (IQR)	7.1 (5.1–10.0)	9.8 (6.8–19.9)	<0.01
Cores per biopsy	Median (IQR)	13 (12–14)	13 (12–14)	0.3
Positive cores per biopsy	Median (IQR)	2 (1–4)	6 (4–8)	<0.01
Percentage of positive cores	Median (IQR)	20 (10–30)	50 (30–70)	<0.01
Highest tumor infiltration per core	Median (IQR)	20 (5–50)	60 (40–80)	<0.01
IHC slides per biopsy	Median (IQR)	4 (2–8)	4 (2–6)	0.06
DRE	non-suspicous	59 (72.8)	44 (42.3)	<0.01
	suspicous	22 (27.2)	54 (51.9)	
cT stage	cT1	58 (71.6)	44 (42.3)	<0.01
	cT2	21 (25.9)	46 (44.2)	
	cT3–4	1 (1.2)	8 (7.7)	
Previous biopsies	Biopsy naive	55 (67.9)	87 (83.7)	0.01
	Repeat biopsy	26 (32.1)	16 (15.4)	
Fusion biopsy	No	53 (65.4)	58 (55.8)	0.2
	Yes	28 (34.6)	46 (44.2)	
Gleason Score at biopsy	6	50 (61.7)	9 (8.7)	<0.01
	7	20 (24.7)	50 (48.1)	
	≥8–10	8 (9.9)	43 (41.3)	
	Unknown GS	3 (3.7)	2 (1.9)	

**Table 3:** Descriptive analyses of 81 patients with positive prostate biopsy, histological positive immunohistochemistry (IHC), and changes in biopsy results due to positive IHC at University Hospital Frankfurt between 01/2017–06/2020. Abbreviations: PCa—prostate cancer; ASAP—atypical small acinar proliferation; RP—radical prostatectomy; RT—radiotherapy; AS—active surveillance; ADT—androgen deprivation therapy; WW—watchful waiting.

Variable	-	Changes in Biopsy Results due to Positive IHC N = 81
Changes in biopsy results due to positive IHC	ASAP to PCa Gleason 6	43 (53.0)
	ASAP to PCa Gleason 7	10 (12.3)
	ASAP to PCa Gleason 8–10	2 (2.5)
	Unilateral PCa to bilateral PCa Gleason 6	10 (12.3)
	Unilateral PCa to bilateral PCa Gleason 7	11 (13.6)
	Unilateral PCa to bilateral PCa Gleason 8–10	4 (4.9)
	Unilateral PCa to bilateral PCa Gleason unknown	1 (1.2)
Changes in biopsy results due to positive IHC	ASAP to PCa	55 (67.9)
	Unilateral PCa to bilateral PCa	26 (32.1)
Therapy after biopsy changes due to positive IHC	RP	42 (51.9)
	RT	7 (8.6)
	AS	27 (33.3)
	ADT/WW	3 (3.7)
	Unknown	2 (2.5)
Pathology at RP in patients with changed biopsy results due to positive IHC	pT2 + Gleason 6	15 (35.7)
	pT3 and/or Gleason 7–10	25 (59.5)
	Regression/unknown	2 (4.8)

## Data Availability

Data will be made available for bona fide research on request.

## Supplementary Materials

The following are available online at https://www.mdpi.com/article/10.3390/curroncol28030197/s1, Table S1: Descriptive analyses of 371 patients with positive prostate biopsy at University Hospital Frankfurt between 01/2017–06/2020, Table S2: Descriptive analyses of 235 patients with negative prostate biopsy at University Hospital Frankfurt between 01/2017–06/2020, Table S3: Descriptive analyses of 290 patients with positive prostate biopsy at University Hospital Frankfurt between 01/2017–06/2020.
