# Supplementary material for: Immunohistochemistry for Prostate Biopsy—Impact on Histological Prostate Cancer Diagnoses and Clinical Decision Making
Source: Curr Oncol. 2021 Jun 9;28(3):2123–33. doi: 10.3390/curroncol28030197 (PMC8293248; doi:10.3390/curroncol28030197)
Supplement: Supplementary file 1 [file curroncol-28-00197-s001.zip › curroncol-1212133-supplementary.pdf]

Current Oncology

MDPI

Article

Immunohistochemistry for Prostate Biopsy—Impact on Histological Prostate Cancer Diagnoses and Clinical Decision Making

Philipp Mandel, Mike Wenzel, Benedikt Hoeh, Maria N. Welte, Felix Preisser, Tahir Inam, Clarissa Wittler, Clara Humke, Jens Köllermann, Peter Wild, Christoph Würnschimmel, Derya Tilki, Markus Graefen, Luis A. Kluth, Pierre I. Karakiewicz, Felix KH. Chun and Andreas Becker

Supplementary Materials

Table S1. Descriptive analyses of 371 patients with positive prostate biopsy at University Hospital Frankfurt between 01/2017–06/2020, stratified according to the performance of immunohistochemistry (IHC). Abbreviations: PSA—prostate-specific antigen; DRE—digital rectal examination; GS—Gleason score.

Variable	-	No IHC N = 163 (43.9%)	IHC N = 208 (56.1%)	*p* Value
Age	Median (IQR)	69 (63–75)	68 62–73)	0.2
Prostate volume	Median (IQR)	43 (30–62)	47 (37–70)	0.09
PSA	Median (IQR)	9.2 (5.7–29.6)	8.2 (5.9–12.9)	0.2
Cores per biopsy	Median (IQR)	13 (12–14)	13 (12–14)	0.6
Positive cores per biopsy	Median (IQR)	6 (4–10)	5 (2–7)	<0.01
Percentage of positive cores	Median (IQR)	50 (30–80)	40 (20–60)	<0.01
Highest tumor infiltration per core	Median (IQR)	70 (50–90)	50 (20–80)	<0.01
DRE	Non-suspicous	64 (39.3)	115 (55.3)	<0.01
	Suspicous	98 (60.1)	86 (41.3)	
cT stage	cT1	64 (39.3)	114 (54.8)	<0.01
	cT2	82 (50.3)	74 (35.6)	
	cT3–4	16 (9.8)	12 (5.8)	
IHC	Negative	-	23 (11.1)	
	Positive	-	185 (88.9)	
IHC slides per biopsy	Median (IQR)	-	4 (2-5)	
IHC slides per biopsy, when IHC negative	Median (IQR)	-	4 (2-6)	
IHC slides per biopsy, when IHC positive	Median (IQR)	-	5 (5-7)	
Positive cores per biopsy, when IHC negative	Median (IQR)	-	5 (2-5)	
Positive cores per biopsy, when IHC positive	Median (IQR)	-	5 (2-7)	
Previous biopsies	Biopsy naive	135 (82.8)	161 (77.4)	0.3
	Repeat biopsy	28 (17.2)	46 (22.1)	
Fusion biopsy	Yes	93 (57.1)	126 (60.6)	0.5
	No	70 (42.9)	82 (39.4)	
Gleason Score at biopsy	6	8 (4.9)	62 (29.8)	<0.01
	7	80 (49.1)	80 (38.5)	
	8–10	73 (44.8)	60 (28.8)	
	Unknown GS	2 (1.2)	6 (2.9)	

Table S2. Descriptive analyses of 235 patients with negative prostate biopsy at University Hospital Frankfurt between 01/2017–06/2020, stratified according to the performance of immunohistochemistry (IHC). Abbreviations: PSA—prostate-specific antigen.

Variable	-	No IHC<br>N = 93 (39.6%)	IHC<br>N = 142 (60.4%)	*p* Value
Age	Median (IQR)	63 (56–68)	63 (57–70)	0.4
Prostate volume	Median (IQR)	60 (40–77)	53 (40–76)	0.3
PSA	Median (IQR)	7.4 (4.7–11.8)	6.5 (4.4–10.8)	0.4
Cores per biopsy	Median (IQR)	12 (12–14)	13 (12–14)	0.01
IHC	Negative	-	142 (100)	
	Positive	-	0 (0)	
IHC slides per biopsy	Median (IQR)	-	4 (2-6)	
Previous biopsies	Biopsy naive	65 (69.9)	97 (68.3)	1
	Repeat biopsy	28 (30.1)	44 (31.0)	
Fusion biopsy	Yes	38 (40.9)	85 (59.9)	<0.01
	No	55 (59.1)	56 (39.4)	

Table S3. Descriptive analyses of 290 patients with positive prostate biopsy at University Hospital Frankfurt between 01/2017–06/2020, stratified according to the performance of immunohistochemistry (IHC) and no change in biopsy results due to IHC performance vs. no IHC performance. Abbreviations: PSA—prostate-specific antigen; DRE—digital rectal examination; GS: Gleason score.

Variable	-	IHC Positive/Negative and No Change in Biopsy Results<br>N = 127(43.8%)	No IHC Performed<br>N = 163 (56.2%)	*p* Value
Age	Median (IQR)	69 (63–74)	69 (63–75)	0.7
Prostate volume	Median (IQR)	45 (36–60)	43 (30–62)	0.4
PSA	Median (IQR)	9.2 (6.6–20.2)	9.2 (5.7–29.6)	0.9
Cores per biopsy	Median (IQR)	13 (12–14)	13 (12–14)	1
Positive cores per biopsy	Median (IQR)	6 (4–8)	6 (4–10)	0.3
Percentage of positive cores	Median (IQR)	50 (30–70)	50 (30–80)	0.5
Highest tumor infiltration per core	Median (IQR)	60 (40–80)	70 (50–90)	<0.01
IHC	Positive	104 (81.9)	0(0)	
	Negative	23 (18.1)	0(0)	
	Not performed	0(0)	163 (100)	
DRE	Non-suspicous	56 (44.1)	64 (39.3)	0.3
	Suspicous	64 (50.4)	98 (60.1)	
cT stage	cT1	56 (44.1)	64 (39.3)	0.5
	cT2	53 (41.7)	82 (50.3)	
	cT3–4	11 (8.7)	16 (9.8)	
Previous biopsies	Biopsy naive	106 (83.5)	135 (82.8)	0.9
	Repeat biopsy	20 (15.7)	28 (17.2)	

Fusion biopsy	Yes	73 (57.5)	93 (57.1)	0.9
	No	54 (42.5)	70 (42.9)	
Gleason	6	12 (9.6)	8 (4.9)	0.5
	7	60 (48)	80 (49.1)	
	8–10	51 (40.8)	73 (44.8)	
	Unknown GS	2 (1.6)	2 (1.2)	
